# Quantitative elemental mapping of biological tissues by laser-induced breakdown spectroscopy using matrix recognition

**DOI:** 10.1038/s41598-023-37258-y

**Published:** 2023-06-21

**Authors:** Patrick Janovszky, Albert Kéri, Dávid J. Palásti, Lukas Brunnbauer, Ferenc Domoki, Andreas Limbeck, Gábor Galbács

**Affiliations:** 1 Department of Inorganic and Analytical Chemistry, University of Szeged, Dóm square 7, Szeged, 6720 Hungary; 2 Institute of Chemical Technologies and Analytics, TU Wien, Getreidemarkt 9/164, 1060 Vienna, Austria; 3 Department of Physiology, University of Szeged, Dóm square 10, Szeged, 6720 Hungary

**Keywords:** Analytical chemistry, Characterization and analytical techniques

## Abstract

The present study demonstrates the importance of converting signal intensity maps of organic tissues collected by laser-induced breakdown spectroscopy (LIBS) to elemental concentration maps and also proposes a methodology based on machine learning for its execution. The proposed methodology employs matrix-matched external calibration supported by a pixel-by-pixel automatic matrix (tissue type) recognition performed by linear discriminant analysis of the spatially resolved LIBS hyperspectral data set. On a swine (porcine) brain sample, we successfully performed this matrix recognition with an accuracy of 98% for the grey and white matter and we converted a LIBS intensity map of a tissue sample to a correct concentration map for the elements Na, K and Mg. Found concentrations in the grey and white matter agreed the element concentrations published in the literature and our reference measurements. Our results revealed that the actual concentration distribution in tissues can be quite different from what is suggested by the LIBS signal intensity map, therefore this conversion is always suggested to be performed if an accurate concentration distribution is to be assessed.

## Introduction

Mineral elements, at the minor and trace concentration levels, have a significant role in or influence on the operation of biological systems^1–3^. In current medical practice and biological research approaches, mineral trace elements are still often determined in mg to grams-sized subsamples of biological material after their homogenization and acid digestion, followed by atomic absorption spectrometry, inductively coupled plasma optical emission spectroscopy (ICP-OES) or inductively coupled plasma mass spectroscopy (ICP-MS) analysis. At the same time, it is well known that spatially resolved information about the trace element distribution in tissues can provide very valuable information about the physiology and pathology of biological systems—this information is lost if bulk analysis is performed.

Several tools of atomic spectroscopy, with various performance, complexity and practicalities, are available today that are able to provide localized microanalysis with μm-range (e.g. 0.1–10 µm) spatial resolution, thus even allowing cellular-level analysis. Some of these techniques are laboratory-based, such as laser ablation ICP-MS (LA-ICP-MS)^4,5^, micro X-ray fluorescence spectroscopy^6^, electron microprobe energy dispersive spectroscopy (EDS)^7^, laser-induced breakdown spectroscopy (LIBS). Some other techniques are either synchrotron-basedsuch as micro particle-induced X-ray emission (PIXE)^8^, synchrotron radiation X-ray fluorescence^8,9^, or harder-to-access, like secondary ion mass spectrometry (SIMS)^7^. These techniques have adequate sensitivity to give rise to at least µg/g level limits of detection in the very small analytical (information) volumes affected by the measurements, but often are limited to heavier elements^10–12^.

In the present context, LA-ICP-MS has received significant attention over the last 10 years and is more and more widely used for the chemical imaging analysis of biological samples^13–16^. The advantage of LA-ICP-MS in this field lies in its low limits of detection (ng/g range) and isotope selective capabilities. Advancements in the field include the use of femtosecond laser pulses to achieve a more stoichiometric ablation^17^, construction of low dispersion ablation cells for improved scanning speed and subcellular spatial resolution^18–20^, construction of novel Peltier element ablation cells that keep the samples at cryogenic temperatures during extended sampling times^21,22^, and development of various imaging software for the visualization and resolution enhancement of elemental maps^23,24^. Although LA-ICP-MS is well on its way to become the standard method of elemental mapping of biological samples, but it still has several practical limitations. One of these is the sequential operation of the most popular quadrupole or sector field mass analyzers –that limits the number of elements that can be analyzed in a single spot. Another mentionable problem is the poor sensitivity of nonmetallic, organogenic elements such as C, H, N, O, F and limited selectivity towards important related elements such as P and S^15^.

LIBS is another laser ablation-based atomic spectroscopy technique, and has distinct advantages over the latter in elemental imaging applications^25,26^. For example, LIBS is capable of working with similar (micrometer-range) spatial (or depth) resolution, but requires no material transport between the laser ablation chamber and the spectrometer (only photons have to travel) and is similarly sensitive to light and heavy elements (detection performance for light elements is not hampered by interferences and elevated background). Single spot and multielement analysis are fully feasible. The fact that LIBS spectra are feature-rich and additionally also contain molecular information helps sample classification of many sample types (e.g.^27–29^), also including biological ones (e.g.^30–32^). It can also be mentioned that for some biologically relevant metals, such as Mg and K, spectral interferences make the ICP-MS determination challenging (e.g. all Mg-isotopes are interferred by C-dimers, whereas ^39^K is interfered by ^38^Ar^1^H); but such interferences do not hamper LIBS measurements. LIBS imaging of various plant, animal and human tissues were already demonstrated to be possible with good sensitivity and spatial resolution (e.g.^33–38^).

Due to their similarities, some technological or methodological results can be transferred from LA-ICP-MS to LIBS (and vice versa), but the fundamental challenges also remain partially common. One such issue is signal calibration for quantitative elemental distribution studies. Quantitative elemental maps obviously provide more and more readily accessible information about the sample than conventional intensity distribution maps, but matrix effects make the accurate calibration difficult^15,33,39,40^. Some novel, innovative approaches have been proposed, but matrix-matched calibration still seems to be unavoidable.

In order to address the above problem, the goal of the present LIBS study, carried out on swine (porcine) tissues as example organic tissues, was to assess the influence of the matrix (tissue type) on calibration plots and to investigate the possibility to use chemometric approaches for the recognition of tissue types in order to help the choice of suitable pre-recorded calibration plots in the conversion of elemental intensity distribution maps to quantitative concentration distribution maps.

## Experimental

### Instrumentation

LIBS experiments were performed using a J-200 LA/LIBS instrument (Applied Spectra) equipped with a 266 nm frequency quadrupled Nd:YAG laser. For every laser shot, the full LIBS spectrum of the sample over the wavelength range from 190 to 1040 nm with a spectral resolution of 0.07 nm were recorded in the Axiom data acquisition software provided by the manufacturer of the instrument. Argon at a flow rate of 1.0 L/min was used as ablation gas. The instrumental performance of the LIBS system was checked on a daily basis using NIST 612 glass standard (National Institute of Standards and Technology, Gaithersburg, MD) to ensure operation according to the specifications. The elemental contents of the porcine (swine) tissue samples were measured at the following wavelengths: Na I 588.99 nm, K I 766.49 nm, Ca II 396.85 nm, Mg II 279.55 nm, Fe I 285.2 nm. When selecting the spectral lines for the elements, the objective was the highest intensity and minimal spectral interference (e.g. see Fig. S1). LIBS instrumental parameters are summarized in Table 1.

**Table 1:** LIBS experimental parameters.

Laser pulse energy	17.5 mJ
Laser spot size	60 μm
Sample stage translation step size	80 µm
Spectrometer gate width	1.05 ms
Spectrometer gate delay	0.5 µs
Ablation gas flow (Ar)	1.0 L/min

For the reference concentration measurement of homogenized and digested tissue samples ICP-OES measurements were carried out using the standard addition calibration strategy. ICP-OES measurements were performed using an iCAP 6500 RAD (ThermoFisher Scientific) spectrometer. The applied viewing height was set to 16 mm. The used ICP-OES instrumental parameters were the followings: coolant gas flow (Ar) was set to 12.0 L/min, auxiliary gas flow (Ar) was set to 0.8 L/min, the nebulizer gas flow (Ar) was 0.7 L/min. During the measurements 0.8 s dwell time (exposure) time was used. A V-groove nebulizer, a glass cyclonic spray chamber with a riser tube and a torch injector tube with 2 mm inner diameter was applied. The sample flow rate was 0.6 mL/min. Background-corrected signals were recorded in three-fold replicates, using an analysis time of 5 s per replicate.

For the homogenization of the tissue samples, a Vortex-Geni 2 mixer, a Bosch MSM 2620B blender, a Heidolph “Hei-Standard” heated magnetic stirrer and a Bandelin Sonorex Super ultrasonic bath were used. Thin sections of the tissues were prepared by using a Thermo Scientific CryoStar Nλ 50 (ThermoFisher Scientific) cryogenic cutting device.

We used LIBS signal normalization by employing the intensity of the C I 247.8 nm spectral line.

### Sample preparation and analysis

In this work, six different domestic swine (*Sus scrofa domesticus*) tissue types (liver, brain, kidney, heart, lung and skeletal muscle) were used as biological matrices, to study the matrix effect on the analytical signal of LIBS. The tissue samples were generally obtained from commercial sources, but brain tissue samples were provided by the research laboratory of the Physiology Department of the University of Szeged. No live animals were involved in this study.

Ultra-pure water (resistivity 18.2 MΩ cm) dispensed from a Barnstead Easypure II water system (ThermoFisher Scientific) was used for all experiments. Merck Emsure trace analytical pure 65% HNO_3_ and 30% H_2_O_2_ were used during the digestion of the swine (porcine) tissue samples. During the standard addition, Merck Emsure trace analytical pure reagents were used. For calibration purposes, the ICP-MS Alfa Aesar Specpure 1000 mg/L S and Merck Certipure „ICP Multi-element standard solution VIII” standards were adequately diluted. Silicon wafers (Infineon Technologies Austria AG) were found to provide optimal properties as carrier material for elemental analysis of tissue cryo-cuts.

Spiking and preparation of the tissue samples were done the following way. In the first step, a commercial blender (merged in 1% HNO_3_ solution for two hours and rinsed thoroughly every time before use) and a vortex homogenizer were employed, in order to destroy fibres and improve on homogeneity. After spiking, vortex homogenization was used. Finally, frozen pellets were prepared from the samples using liquid nitrogen and stored in a freezer at − 70 °C until further use.

Thin sections of brain tissue with 30 µm thickness were prepared prior to LIBS imaging at − 70 °C. The sections were then deposited onto silicon wafers. A gentle vacuum was applied to facilitate the removal of some of the water content. For easier handling and transportation of the samples, the silicon substrates were attached to glass microscope slides with double-sided foamed adhesive tape.

Prior to all ICP-OES measurements, acidic digestion was applied to approximately 0.25 g sample mass, using a mixture of 1.5 mL cc. HNO_3_ and 0.5 mL H_2_O_2_ for two hours, promoted by sonication until clear solutions were obtained. The analysis was performed on 0.25 mL digested sample volume diluted to 9.0 mL by using a mixture of 0.25 mL cc. HCl and 8.5 mL 1 v/v% HNO_3_, performed in three repetitions.

Validation of LIBS-determined concentrations shown in "Quantitative elemental mapping of brain tissue" section (Fig. 4) for the brain tissues was done by performing ICP-OES analysis following acid digestion, according to the above recipe, of the grey and white matter in brain tissues. This also allowed us to correct LIBS calibration plots obtained for homogenized brain tissue in order to obtain separate calibration functions for the two grey and white matter. We also determined the wet-to-dry weight ratios, which could be used towards assessing the matrix effect. Please find these analytical data in Table S1.

### Data evaluation

Spectral line identification was carried out using version 18.0 of the Clarity software (Applied Spectra, USA) of the LIBS instrument. Linear discriminant analysis (LDA) and visualization of LIBS elemental maps were carried out by in house developed codes written in *R* programming language, using OpenImageR and MASS packages.

## Results and discussion

### Assessment of matrix effects

In order to assess the influence of different tissue types on calibration plots, calibration was performed in six different swine organ tissue types (lung, heart, kidney, liver, skeletal muscle and brain). These tissue types were chosen to represent a relatively wide range of cell types, water and lipid content, density, etc. LIBS signal intensities for the spectral lines of the following elements were measured: K, Na, Mg, Ca, Fe. Signal normalization to the C I 247.8 nm line intensity was employed considering that carbon is one of the main components in organic tissues. The actual concentration of these elements in the spiked samples were determined by ICP-OES, following acid digestion in a HNO_3_/H_2_O_2_ mixture. All calibration standards and samples were presented as thin sections, in order to ensure an as similar as possible laser ablation behaviour.

**Figure 1:**
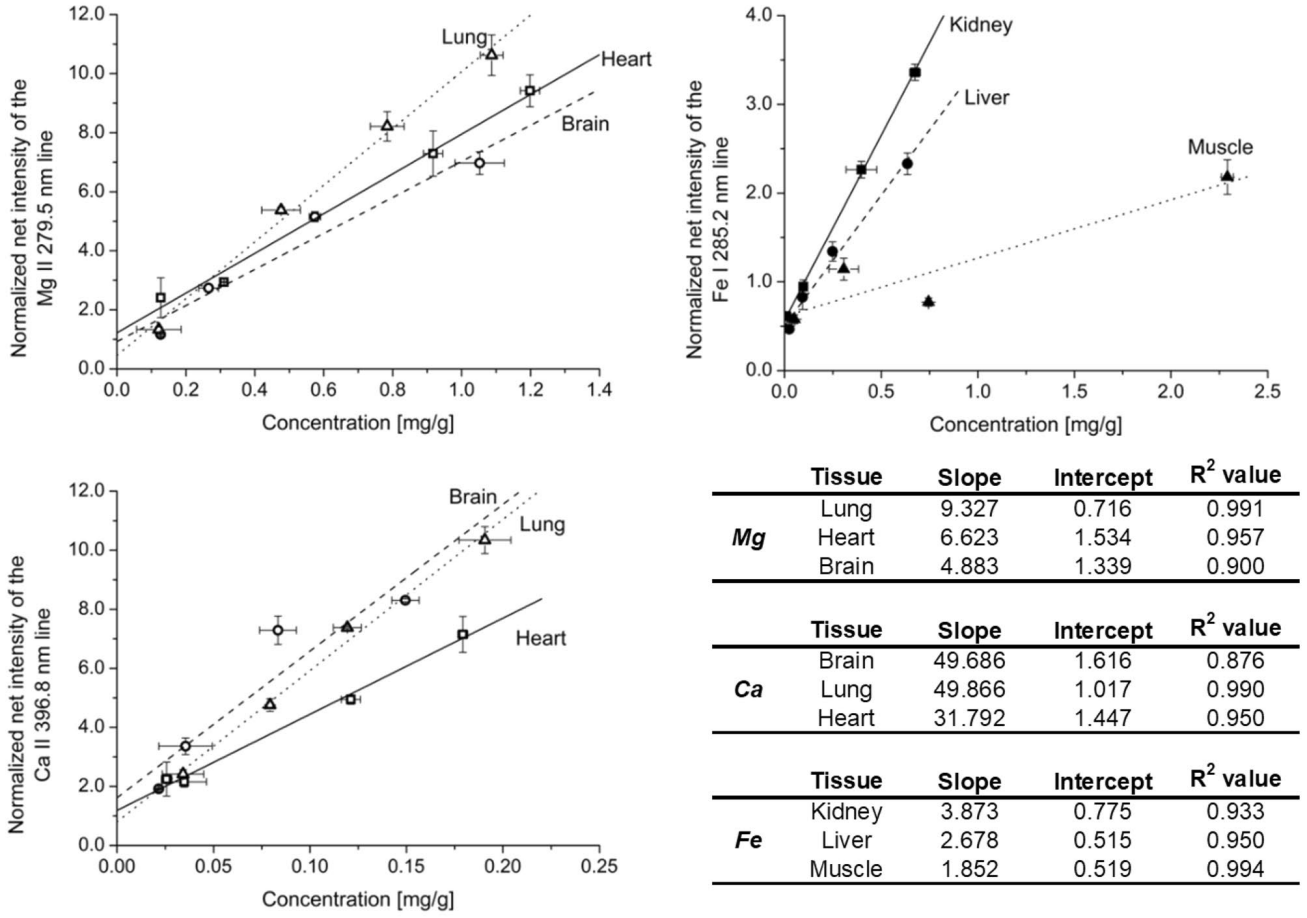
LIBS calibration plot examples obtained for Mg, Fe and Ca in different swine (porcine) organ tissue matrices. Samples were spiked with standards and their concentration were determined by reference methods. LIBS signal normalization to the C I 247.8 nm lines was employed. All measurements were performed in three replicates.

The findings are demonstrated in Fig. 1 for four selected elements and some of the tissue types. The first data points in the plots represent the unspiked samples. As it can be seen, the natural concentration of these elements in the samples is in the 0.02–2 mg/g range and the plots are reasonably linear. Please note that although some effort was done to adjust the spiked amounts according to the natural concentration of the elements, but the actual slope of the calibration plots (irrespective of the matrix) is not directly informative. Comparison of the slopes is only meaningful between different matrices. As expected, a strong matrix effect was found for most elements (spectral lines), but in general the situation is quite complex. The slope of the calibration lines were several times larger for certain element/matrix combinations than for others (e.g. observe the case of Fe in kidney and liver versus muscle), whereas in other cases, the slope was reasonably similar in all matrices (e.g. Mg in heart, lung and brain).

The above observations underline the importance of matrix-matched calibration in quantitative LIBS analysis of biological tissues. In our opinion, there is no real alternative to the approach which is based on the use of matrix-matched, carefully homogenized calibration standards prepared by spiking.

### Matrix recognition

Localized quantitative LIBS analysis of biological samples is possible by using matrix-matched calibration. Quantitative elemental mapping is therefore theoretically also possible, if there is only one tissue type present in the thin sectioned sample. It has to be mentioned though that this analytical task requires the a priori knowledge of the tissue (matrix) type in order to allow for the selection of the correct calibration data from pre-recorded data sets. In such cases however when inadequate information is available about the tissue type or when more than one tissue type is present within the section (this can easily occur in sections with a larger surface area), the automatic recognition of tissue types would be very practical.

Matrix type discrimination based on their LIBS spectra has already been proposed in the literature, with the intention of directing a surgical laser scalpel by differentiating between tissue types in localized analysis^41,42^ or generating qualitative discrimination maps for industrial samples, such as plastics^43^. At the same time, to the best of our knowledge, the qualitative discrimination capabilities of LIBS were not attempted before to be used to support quantitative elemental mapping (in other words: to convert intensity distribution maps to concentration maps) in organic tissues. In the present study, we also assessed this possibility. Here, we used the same six swine (porcine) tissue types as earlier, but we separated brain tissue into two sub-types which are known as “white” and “grey” matter, which can generally be distinguished under the microscope by their color. Grey matter *(substantia grisea)* is chiefly made up of neuronal and glial cell bodies, while white matter (*substantia alba*) is composed of nerve fibers and their lipid-rich myelin sheaths^44–46^.

**Figure 2:**
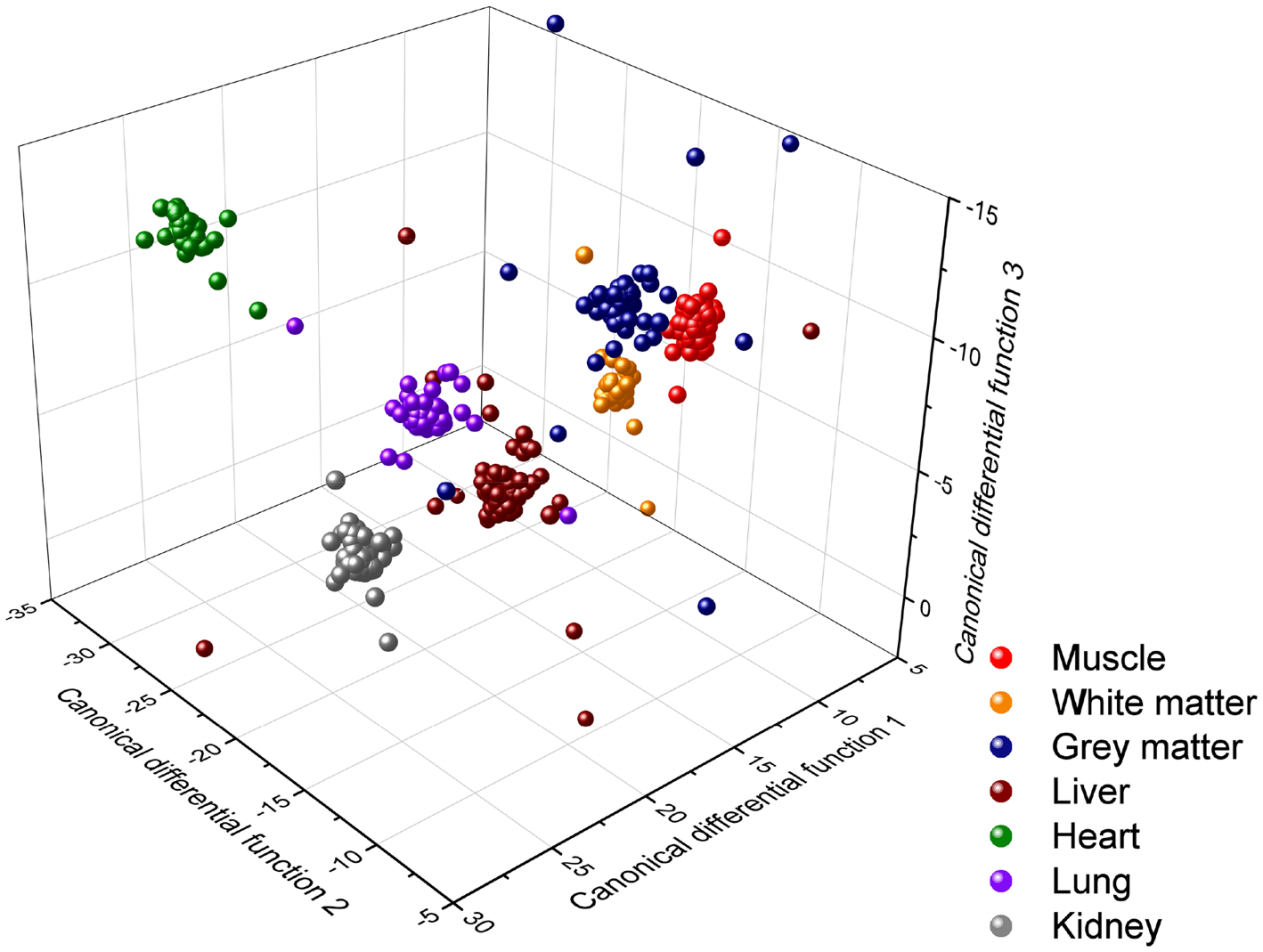
Visual representation of LDA classification results of the studied seven organic tissue types, in the sub-space of the first three canonical differential functions.

As the first step, supervised classification of the now seven tissue types was carried out using multi-class linear discriminant analysis (LDA) of their LIBS spectra. The spectra were scaled again based on the intensity of the C I 247.8 nm spectral line. The model was based on 200 observations (spectra) for each tissue type. As it can be seen in Table 2 and Fig. 2, the discrimination was quite successful, as on average, the model predicted the tissue type with better than 98% accuracy. Validation of the results was performed by repeating the modelling by using a randomly selected two-third of the data set five times, which resulted in 95% accuracy with only 1% scatter. Kidney and muscle tissues were found to be very distinct from the rest and classified correctly in 100% of the cases. On the other hand, liver spectra were incorrectly classified, although in a small number of cases, into five other groups.

**Table 2:** Confusion matrix (with values as percentages) for the LDA classification of the studied seven organic tissue types.

	Original group						
	Grey matter (%)	White matter (%)	Heart (%)	Kidney (%)	Liver (%)	Lung (%)	Muscle (%)
Predicted group							
Grey matter	**98**	0	0	0	0	0	0
White matter	0	**99**	0	0	1	0	0
Heart	0	0	**98**	0	1	2	0
Kidney	0	0	0	**100**	1	0	0
Liver	0	1	2	0	**95**	0	0
Lung	0	0	0	0	1	**98**	0
Muscle	2	0	0	0	1	0	**100**

**Figure 3:**
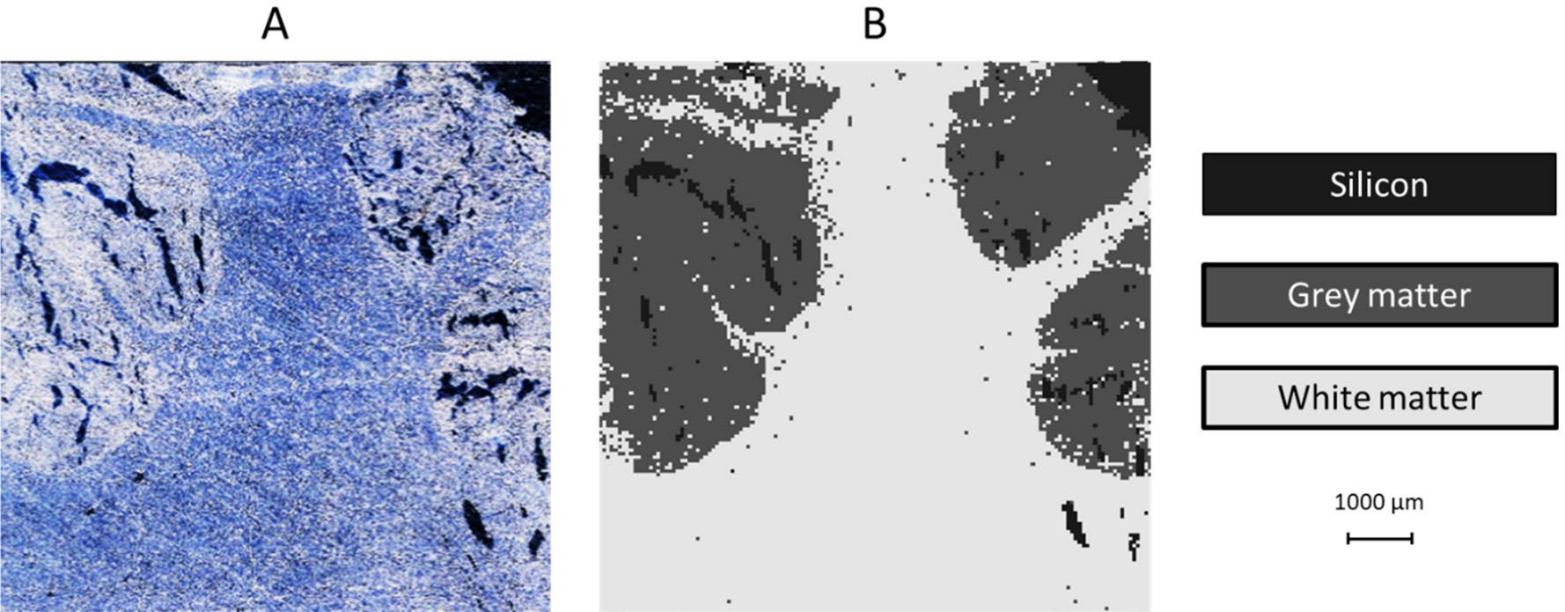
Automatic pixel-by-pixel local organic tissue recognition in a LIBS image, based on LDA classification. The sample was a thin section of a brain tissue (cerebral cortex) presented on a silicon chip. (**A**) Microscope image (taken before LIBS mapping), (**B**) LIBS image with tissue-dependent pixel colors.

Next, we applied the discrimination power of LDA to the spectra collected in each of the individual pixels of a LIBS imaging data set. The test sample was a 30 µm thin section of a brain tissue (ca. 9.3 × 9.3 mm) affixed to a silicon chip. Within the scanned area of this sample, both the grey and white brain tissues were present, as well as areas of the silicon substrate uncovered by the organic tissue (please see the microscope image in Fig. 3a). This presented a three-class discrimination scenario. LDA was applied in two steps; in the first stage, the bare Si areas (pixels) were discriminated, whereas in the next stage, the two tissues were distinguished. The LIBS image were then colored pixel-by-pixel according to the result of the tissue type classification. The resulting discrimination map can be seen in Fig. 3b. As it can be appreciated from the images, the LIBS image represents the microscope image (actual tissue structure) with good fidelity; only a small number of sporadic misclassifications are present. In some areas, the fine structure (especially the “torn” or see-through parts) of the tissue appear to be somewhat smaller than in the microscope image—this is simply due to that (a) the spatial resolution of the LIBS map is poorer than that of the microscope image, and (b) small distortions due to the ablation of the thin organic sample may occur.

In our opinion, this experiment demonstrates well the feasibility of automatically recognizing the tissue type in each pixel of a LIBS image with very good accuracy. This allows for the automatic use of the proper matrix-matched calibration data for the generation of quantitative elemental maps. Adaptation of the procedure to other tissue types or more tissue sub-types within the sample can be done with relative ease. It can also be added that it is a pre-requisite of the matrix-recognition procedure based on LDA, or any other supervised classification method, that the list of potential tissue types present in the sample section to be known a priori. However, this cannot be considered as a limitation, since in biological/medical studies the sampled organ is always known, thus a list of potential tissue types (e.g. sub-type and adjacent tissue types) can be assembled and their representative LIBS spectra required for the construction of the chemometric model can be collected.

### Quantitative elemental mapping of brain tissue

**Figure 4:**
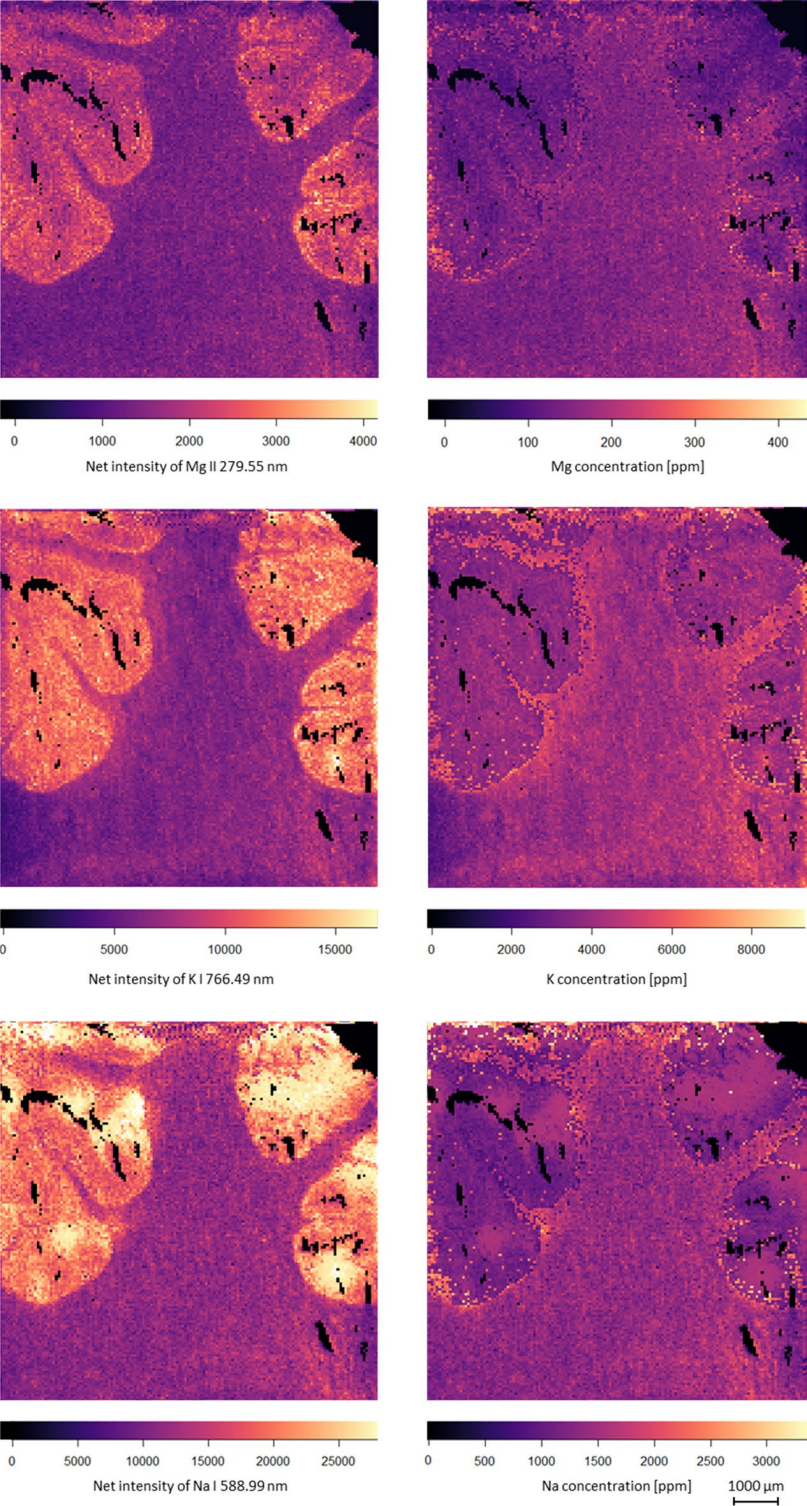
A comparison of LIBS intensity maps (left column), and matrix-corrected concentration maps (right column) for elements Mg, K and Na in the same brain section sample shown in Fig. 3.

We performed the complete procedure for the conversion of LIBS elemental signal intensity maps to quantitative concentration maps proposed above on the same brain tissue sample data set used previously. The calculations were done for elements Mg, K and Na. We chose these elements as the subject of our study because recently imbalances in alkali and alkali earth ion levels (particularly Mg^2+^, K^+^ and Na^+^) in the brain have been associated with some neurodegenerative disorders such as Alzheimer’s disease, Parkinson’s disease or multiple sclerosis^47–49^. It is also known that the concentration of Na^+^, K^+^ and Ca^2+^ is altered in and around tumours, which finding has led to research into new approaches towards cancer diagnostics^50^. The matrix-matched calibration procedure described in "Assessment of matrix effects" section was expanded by handling the white and grey material as separate tissues and the corresponding intensity—concentration proportionality was used to produce the concentration maps shown in Fig. 4. Each individual pixel in the map was categorized as grey or white matter (or Si substrate) according to our LDA-based tissue recognition described in "Matrix recognition" section.

The most striking feature of the concentration maps is that they reveal that the concentration distribution of the elements between the two brain tissue types is quite different from what is suggested by the intensity maps. While the intensity maps suggest that elemental concentrations should be higher in the grey matter than in the white matter, the concentration maps reveal that actually the concentrations are quite similar; the difference is only 1 to 5%. In our opinion, the reason for this lies in the fact that the water content of the two tissues are significantly different, as is described in the medical literature (e.g.^44–46^). This was also confirmed by our measurements: for grey matter, we measured a wet-to-dry weight ratio (also known as the reduction factor) of 6.20, whereas for white matter it was only 4.37. It is also known that white matter has almost 50–70% more lipid content than grey matter. We also performed plasma temperature calculations using the Saha-Boltzmann plot method based on argon spectral lines (utilizing that all LIBS experiments were done under argon flow). These calculations yielded T_white_ = 20,700 K and T_grey_ = 18,700 K, thereby numerically also demonstrating the effect of matrix on the plasma. These differences in the matrix cause different signal suppressions, therefore LIBS intensity maps can suggest a concentration contrast higher than in reality. The concentration range shown in our concentration maps for the white and grey matter also match fairly well the literature values (e.g.^46^) and we also cross-checked them by ICP-OES analysis (please see description of procedure in "Sample preparation and analysis" section. and the results in Table S1). Based on the above, we would like to emphasize that matrix-matched pixel-by-pixel calibration of LIBS intensity maps are crucial if the correct assessment of concentration distributions is targeted, as the simple assumption that intensity maps correctly represent concentration distributions can lead to false diagnostic conclusions.

We would also like to point out that the “image quality” (sharpness, contrast) of the LIBS intensity maps in Fig. 4 are clearly better than that of LIBS concentration maps. In the latter, some “faulty” pixels can be seen, mostly but not exclusively around the border of white and gray matter areas. This is due to the slightly less than 100% accuracy of the matrix recognition process, which therefore occasionally mistakes the tissue type for a pixel. However, logically these small faults occur mainly in the zone where the two tissue types are mixing with each other for real within the focal spot of the laser represented by a pixel in the map. Thus, it is understood that intensity maps are better for tissue visualization, but concentration maps should be preferred for representing the concentration distribution.

## Conclusions

Using the example of six types of porcine (swine) organic tissue, we demonstrated that LIBS signal intensities suffer from a strong matrix effect caused by the organic tissues, therefore the conversion of LIBS intensity maps to concentration maps is only feasible if matrix-matched external calibration is performed. We have shown that such a calibration can be supported by a pixel-by-pixel recognition of the matrix (organic tissue type) in the LIBS hyperspectral map. This supervised classification is clearly possible in a medical (or veterinarian) situation, since an appropriate machine learning algorithm can be trained by spectra collected from just a few locations in the sectioned sample which were recognized by a qualified personnel. On a brain (cerebral cortex) sample, we successfully performed this matrix recognition with a very good accuracy (around 98%) by employing linear discriminant analysis of the collected LIBS hyperspectral data set. By combining the information from a stored matrix-matched external calibration curve and the tissue distribution map, we have converted a LIBS intensity map to a correct concentration map. This also revealed that the actual concentration distribution can be quite different from what is suggested the intensity map, therefore this conversion is always suggested to be performed if an accurate concentration distribution within a sectioned organic tissue sample is to be assessed.

We would also like to mention that calibration free LIBS (CF-LIBS^51^ is another possibility to obtain quantitative elemental distribution maps by LIBS, which has the advantage of not being limited to the availability of matrix-matched calibration standards. However, CF-LIBS is known to provide a quite limited accuracy for minor and trace elements and its reliability is also dependent on several experimental conditions^52^. As we wanted to present an analytical approach that is applicable to all elements and all concentration ranges, we used external calibration combined with matrix recognition.

## Supplementary Information

Supplementary Information.

## Data Availability

The datasets used and/or analysed during the current study are available from the corresponding author on reasonable request.
