# Supplementary material for: Quantitative elemental mapping of biological tissues by laser-induced breakdown spectroscopy using matrix recognition
Source: Sci Rep. 2023 Jun 21;13:10089. doi: 10.1038/s41598-023-37258-y (PMC10284993; doi:10.1038/s41598-023-37258-y)
Supplement: Supplementary file 1 — Supplementary Information. [file 41598_2023_37258_MOESM1_ESM.docx]

**Supplementary data**

**Fig S1.** Illustrative LIBS spectra of swine (porcine) tissues in the 391 to 400 nm range, in the vicinity of Ca II 393.4 nm and Ca II 396.85 nm spectral lines.


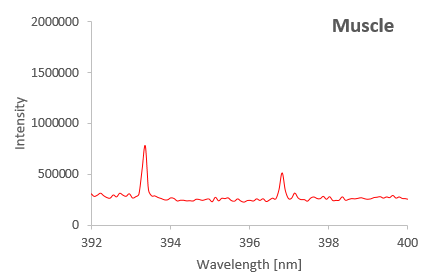

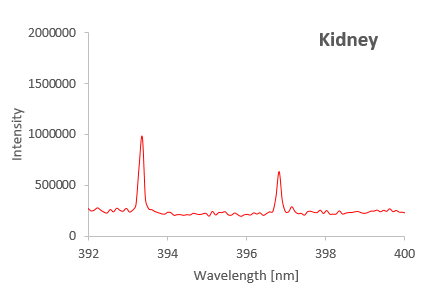


**Table S1.** Comparison of wet swine brain tissue (grey and white matter) concentration for Mg, K and Na, as determined by ICP-OES following acid dissolution and by LIBS quantitative elemental mapping. Data are given as averages based on three parallel analysis (for ICP-OES) and as pixel averages. Values in brackets are standard deviations.

|  | **Mg conc. [ppm]** | **K conc. [ppm]** | **Na conc. [ppm]** |
| --- | --- | --- | --- |
| White matter (ICP-OES) | 138 (2) | 3594 (73) | 1302 (29) |
| Grey matter (ICP-OES) | 132 (2) | 3738 (29) | 1281 (39) |
| White matter (LIBS). | 126 (28) | 3654 (609) | 1419 (287) |
| Grey matter (LIBS). | 117 (28) | 4479 (911) | 1167 (257) |
